# Transmission Risk Predicting for Schistosomiasis in Mainland China by Exploring Ensemble Ecological Niche Modeling

**DOI:** 10.3390/tropicalmed8010024

**Published:** 2022-12-28

**Authors:** Jingbo Xue, Xiaokang Hu, Yuwan Hao, Yanfeng Gong, Xinyi Wang, Liangyu Huang, Shan Lv, Jing Xu, Shizhu Li, Shang Xia

**Affiliations:** 1 NHC Key Laboratory of Parasite and Vector Biology, National Institute of Parasitic Diseases, Chinese Center for Disease Control and Prevention (Chinese Center for Tropical Diseases Research), WHO Collaborating Center for Tropical Diseases, National Center for International Research on Tropical Diseases, Shanghai 200025, China; 2 School of Global Health, Chinese Center for Tropical Diseases Research, Shanghai Jiao Tong University School of Medicine, Shanghai 200025, China; 3 Hainan Sub-Center of Chinese Center for Tropical Disease Research, Hainan Tropical Diseases Research Center, Haikou 571199, China

**Keywords:** *Schistosoma japonica*, snail habitat, ecological niche modeling

## Abstract

Schistosomiasis caused by *Schistosoma japonicum* is one of the major neglected tropical diseases worldwide. The snail *Oncomelania hupensis* is the only intermediate host of *S. japonicum,* which is recognized as an indicator of the schistosomias occurrence. In order to evaluate the risk of schistosomiasis in China, this work investigate the potential geographical distribution of host snail habitus by developing an ensemble ecological niche model with reference to the suitable environmental factors. The historical records of snail habitus were collected form the national schistosomiasis surveillance program from the year of 2005 to 2014. A total of 25 environmental factors in terms of the climate, geographic, and socioeconomic determinants of snail habitats were collected and geographically coded with reference to the snail data. Based on the correlations among snail habitats and the geographically associated environmental factors, an ensemble ecological niche model was developed by integrating ten standard models, aiming for improving the predictive accuracy. Three indexes are used for model performance evaluation, including receiver operating characteristic curves, kappa statistics, and true skill statistics. The model was used for mapping the risk of schistosomiasis in the middle and lower reaches of the Yangtze River. The results have shown that the predicted risk areas were classified into low risk (4.55%), medium risk (2.01%), and high risk areas (4.40%), accounting for 10.96% of the land area of China. This study demonstrated that the developed ensemble ecological niche models was an effective tool for evaluating the risk of schistosomiasis, particularly for the endemic regions, which were not covered by the national schistosomiasis control program.

## 1. Introduction

Neglected tropical diseases (NTDs) are a diverse group of diseases that are mainly prevalent in tropical areas, where they mostly affect impoverished communities [1]. Many of NTDs are vector-borne, have animal reservoirs, and their epidemiology is complex and often related to environmental conditions and climate changes. In 2015, the United Nations set up the 2030 Agenda for Sustainable Development by adopting the 17 Sustainable Development Goals (SDGs), which call for urgent action to deal with the impacts of climate change to successfully achieve all Sustainable Development Goals (SDGs) [2]. Goal 3 of SDGs has targeted the end of the epidemics of NTDs by 2030. In doing so, WHO launched its road map for NTDs entitled “Ending the neglect to attain the Sustainable Development Goals: a road map for NTDs 2021–2030” [3].

Schistosomiasis is one of the NTDs, which is prevalent in 78 countries in tropical and subtropical areas of the world. Current global infections are estimated at 240 million people, with more than 700 million people at risk for infection [4]. The transmission of schistosomiasis is associated with its intermedia host snail, the habitat of which is affected by a series of climate environments, including meteorological, geographical, and ecological factors. In China, *Schistosoma japonicum* is the main endemic species. Because of its wide geographic distribution and large clinical case burden, *S. japonicum* is a major public health and socioeconomic concern [5]. Since the initiation of the national schistosomiasis control program in the 1950s, considerable progress has been made toward the control of *S. japonica* in China [6,7]. China is moving toward the interruption of transmission and elimination of schistosomiasis across the country by 2030 [8]. By the end of 2021, among the 12 endemic provinces (municipality and autonomous region) for schistosomiasis in China, five of them, i.e., the Shanghai, Zhejiang, Fujian, Guangdong, and Guangxi, have maintained the criteria of elimination, the Sichuan and Jiangsu provinces achieved the goal of transmission interruption, and the Yunnan, Hubei, Anhui, Jiangxi and Hunan provinces maintained the criteria of transmission control [9].

Currently, schistosomiasis is characterized by low prevalence and low infection intensity in areas where transmission is ongoing. The climate environment and socioeconomic factors associated with intermediate host snail distribution, however, remain unchanged [10]. Consequently, several challenges remain for the interruption of transmission and eventual elimination of schistosomiasis in China. These challenges include the management of infected humans and livestock, a high seropositive infection rate in humans in endemic areas, and host snail persistence in areas populated with infected animals [11,12]. Furthermore, currently available detection and monitoring tools are inadequate for optimal control of schistosomiasis, resulting in an underestimate of incidence, which increases the risk of schistosomiasis re-emergence [13]. A targeted assessment of schistosomiasis prevalence is therefore needed to facilitate progress toward elimination.

Transmission of schistosomiasis is associated with variations in climate environments, and climate changes will affect the spatial distribution and transmission intensity of infection [14]. Transmission is also indirectly affected by population movement and socioeconomic development. These indirect risk factors and climate environmental changes impact disease incidence and management. Examination of correlations between the climate and social environment with the occurrence of schistosomiasis will provide a better understanding of schistosomiasis transmission. In this study, climate change factors, together with geographic and socioeconomic factors, were evaluated in ecological niche models that assessed schistosomiasis occurrence. In this manner, areas at high risk of infection and transmission were identified, and with identification it will be possible to evaluate the impact of climate changes and to implement schistosomiasis control and elimination programs in China.

Ecological niche modeling integrates species point prevalence data with environmental raster data to estimate the ecological suitability of vector distribution, thereby predicting the actual or potential distribution of infection [15]. Ecological niche modeling has been widely employed in infectious disease research [16] and it is useful for the analysis of associations among environmental risk factors and infection prevalence as a means by which to predict disease transmission [17,18]. To date, ecological niche modeling has been used to predict the potential habitats of *Oncomelania hupensis* (the intermediate host of *S. japonicum*) [19,20,21] and to predict schistosomiasis vectorial capacity based on future climate scenarios [22]. Previous studies have successfully used ecological niche modeling for the prediction of schistosomiasis transmission, but few of these studies have assessed the direct risk of schistosomiasis transmission by ecological niche modeling. In this study, an ensemble ecological niche modeling approach was developed to extract the ecological elements within endemic areas of schistosomiasis that predict potential risk for schistosomiasis in China.

## 2. Materials and Methods

### 2.1. Ethics

Ethics issues were not relevant to this study.

### 2.2. Study Area

**Figure 1:**
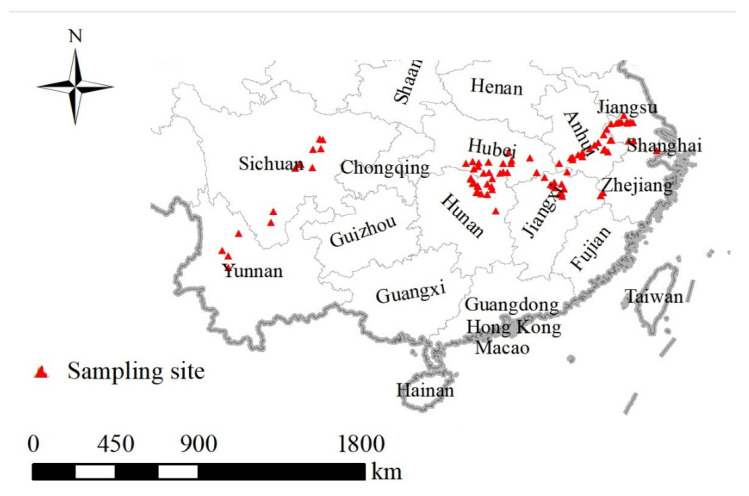
The geographical locations of historical host snail records. A total of 92 villages were selected as the sample sites due to their reported cases of schistosomiasis during the years 2005 to 2014. The snail records in each sample site were obtained from the National Schistosomiasis Surveillance Program.

Villages that reported schistosomiasis cases during the years 2005 to 2014 were selected for analysis. In China, the national snail survey program is completed annually to understand snail distribution and density, as well as the environmental characteristics of snail habitats. Snail survey data for 92 villages at surveillance sites were obtained from the national schistosomiasis surveillance database with the exclusion of repeated and incomplete data. The latitude and longitude for each village were determined (Figure 1).

### 2.3. Environmental Factors

#### 2.3.1. Environmental Dominants of Snail Habitus

The distribution of snails is related to geographical and ecological factors that include submersion time during the flooding season, groundwater level, soil moisture, vegetation, and light intensity. Prolonged submersion, high groundwater levels, and moist soil increase snail density. Suitable snail habitats require a mild climate and abundant rainfall. Conditions need to provide an annual average temperature of over 14 °C and an annual average precipitation of over 750 mm. Potential ecological predictors for host snail distribution of schistosomiasis were identified from the existing literature. The geographical distribution of snails in marshlands and lake regions is related to ecological factors, including elevation, soil type, rainfall pattern, proximity to human and livestock areas, and climate factors related to temperature and precipitation. A series of environmental factors related to the distribution of snail habitus can be divided into four categories, including meteorological, bioclimatic, geographical, and socioeconomic variables.

#### 2.3.2. Data Processing

As summarized in Table 1, a total of 25 environmental factors, in terms of climate, geographical, and socioeconomic factors, were included in the environmental dataset. Specifically, climate factors were extracted from the Chinese Meteorological Background dataset based on the meteorological data from 1950 to 1990, with a 500 m by 500 m spatial resolution (six factors), created by the Resource and Environmental Science Data Center of the Chinese Academy of Sciences (http://www.resdc.cn, accessed on 1 October 2022). The global climate dataset was based on the meteorological data of China from 1955 to 2000, with a 1 km by 1 km spatial resolution (19 bioclimatic factors) using WorldClim v. 2.0 (http://www.worldclim.org, accessed on 10 September 2022). Geographical factors with a 1 km by 1 km spatial resolution included elevation (data from 2000), landform (data from 2005), land use (data from 2005), soil texture (data from 1995), and annual NDVI (data from 2004 to 2015) (ANDVI). Elevation, soil texture, and ANDVI data were obtained from the Resource and Environmental Science Data Center of the Chinese Academy of Sciences. Landform and land use data were downloaded from the National Earth System Science Data Center (http://www.geodata.cn, accessed on 20 October 2022). Socioeconomic factors were derived from 2010 data, including the density of the bovine population, gross domestic product, and human population density. Bovine population density data with a 10 km by 10 km spatial resolution were captured by the Food and Agriculture Organization of the United Nations (http://www.fao.org, accessed on 11 August 2022), while gross domestic product and population density data with a 1 km by 1 km spatial resolution were downloaded from the Resource and Environmental Science Data Center of the Chinese Academy of Sciences (http://www.resdc.cn) (accessed on 10 October 2022).

The values of each environmental factor were clipped to a standard base map of China in ArcGIS Version 10.6 (U.S. ESRI, Redlands, CA, USA) using the nearest neighbor resampling algorithm (for categorical factors) or a bilinear resampling algorithm (for continuous factors), with a 1 km by 1 km spatial resolution.

Collinearity diagnostics were carried out on all environmental factors, except for two categorical variables (land use and landform). Collinearity was assessed using Pearson’s correlation coefficient in Software Version 4.2.2 (U.S. EPA, Washington, DC, USA) with pairwise correlation coefficients and a correlation coefficient matrix. An absolute correlation coefficient of >0.90 represented a strong degree of correlation, with a value of zero indicating no correlation. Collinear variables were removed based on biological significance, and the remaining environmental variables were included in the ecological niche models.

The importance of environmental factors of the ensemble model was analyzed by using the variable importance calculation function of the biomod2 package. The principle was to shuffle a single data variable and then assess the predictive models with the “shuffled” dataset. A simple Pearson’s correlation between the reference prediction and the “shuffled” prediction was computed to measure the contribution rate of the variable to the model. The contribution rate of the environmental factors to the ensemble model was assessed with the biomod2 package. The percentage of different environmental factors was calculated and sorted to identify the main influencing factors.

### 2.4. Ensemble Ecological Niche Modeling

#### 2.4.1. Standard Ecological Niche Models

Ecological niche models (ENMs) that were originally developed for ecological and conservation purposes are used increasingly to model spatial distribution and potential risk of occurrence for a range of diseases and vector species. Ten standard ecological niche models were used to examine associations of *S. japonica* occurrence with environmental, geographic, climate, and socioeconomic risk factors using the biomod2 package [23].

As shown in Table 2, the selected standard models were classified into four categories with reference the mechanism of their prediction mechanisms. They are surface range envelope (SRE) models based on an environmental envelope (threshold-based) algorithm, generalized linear models (GLM), generalized additive models (GAM), and multivariate adaptive regression spline (MARS) models, based on a statistical regression algorithm, generalized boosted models (GBM), classification tree analysis (CTA) models and flexible discriminant analysis (FDA) models, based on a classification algorithm, artificial neural network (ANN), random forest (RF), and maximum entropy (MaxEnt) models, based on a machine learning algorithm.

Models were run using training datasets, with 10 runs completed for repeated models using the same parameters, resulting in the creation of 100 ecological niche models.

#### 2.4.2. Model Evaluation and Validation

Model performance was assessed with test datasets using threshold-independent and threshold-dependent measures. In this study, the most commonly used indices in the biomod2 package, which are, respectively, the receiver operating characteristic (ROC, the threshold-independent index) [24], kappa statistic (threshold-dependent index), and true skill statistic (TSS, threshold-dependent index), were employed to assess the performance of the single and the ensemble ecological niche models [25].

The receiver operating characteristic curve takes the false positive rate as the abscissa and the true positive rate as the ordinate, which is widely used in all types of model prediction accuracy evaluation. The area under the ROC curves (AUC) is used to reflect the ability of the model to distinguish between positive and negative samples, that is, the classification prediction ability of species distribution. The advantage of the ROC curve is that it is not affected by the unbalanced distribution of positive and negative samples in test data sets and the judgment threshold and can stably evaluate the model performance under various data conditions. The value range is from 0 to 1, and the value size represents the accuracy of the model prediction. The closer the value is to 1, the higher the model accuracy is. A value less than 0.5 is generally defined as poor model performance and even not necessarily better than the random prediction result. A value greater than 0.9 is considered excellent prediction performance.

Kappa statistics can evaluate the overall prediction accuracy of the model after random calibration, which is related to the distribution incidence and judgment threshold. Its value is located in the interval from −1 to 1. When the value is less than 0, it indicates that the prediction result of the model is not a strong and random prediction. The closer the value is to 1, the more consistent the prediction result of the model is with the actual observation result and the better the accuracy of the model is. A value greater than 0.85 is considered a very good model performance.

TSS value is an improved index based on kappa statistics. Its result is not affected by the incidence of distribution but by the judgment threshold, and it can make an accurate judgment on the accuracy of the model. Its value range is as same as kappa. When the TSS value is greater than 0.85, it can be considered that the prediction results of the model are ideal.

All three of these indices may be used differently to evaluate the prediction accuracy of ecological niche models. ROC statistics are used to assess the ability to distinguish the presence or absence of species distribution of the model, while kappa statistics and TSS measure the consistency between model prediction results and sample data [26]. Using a combination of these three indices more accurately evaluates the predictive performance of 10 ecological niche models and yields an optimal model. Table 3 shows the assessment criteria for the prediction accuracy of ecological niche models.

To evaluate the developed models for snail-suitable habitat prediction, the ground truth of the ecological niche modeling outputs was conducted by comparing the suitable distribution of the snail population. Locations within the study area predicted different habitat suitability values. Specifically, 25% of the total ground survey snail distribution sites were selected as a validation dataset, which was used for comparison of model-predicted suitable habitats. The ratio of such a comparison was interpreted as the accuracy of the developed prediction model.

The model accuracy in terms of ROC, KAPPA, and TSS values was examined by the standard deviations (X ± s), and the difference between these indexes was compared using the Kruskal-Walis H test, a non-parametric test based on rank order, with a hypothesis test level of α = 0.05.

#### 2.4.3. Ensemble Model and Risk Classification

Due to the uncertainty of predictions made using a basic ecological niche model, an ensemble modeling approach was created by integrating the developed basic models to improve the predictive accuracy of schistosomiasis transmission risk. The inclusion of ecological niche models to build an ensemble model was based on a ROC threshold value of >0.90 and a TSS value of >0.85. The prediction results of each ecological niche model were normalized to account for raster data values ranging from 0 to 1. Following the definition of the weight according to TSS values, prediction results of the ensemble model were estimated using a weighted average method.

Schistosomiasis transmission risk among different regions was evaluated according to four classifications based on the prediction of the ensemble model.

These included no-risk areas, low-risk areas, medium-risk areas, and high-risk areas of transmission risk that were predicted by the ensemble model identified at a minimum presence threshold.

- No-risk area: a presence probability ranging from 0 to the minimum presence threshold,
- Low-risk area: a presence probability ranging from the minimum presence threshold to 0.70,
- Medium risk area: a presence probability ranged from 0.70 to 0.80,
- High-risk area: a presence probability of 0.80 to 1.00.

Geostatistical analysis of the distribution of areas at risk for schistosomiasis transmission was carried out in 12 endemic provinces, and the area proportion was calculated for various levels of risk.

## 3. Results

### 3.1. Results of Collinearity Diagnostics

**Figure 2:**
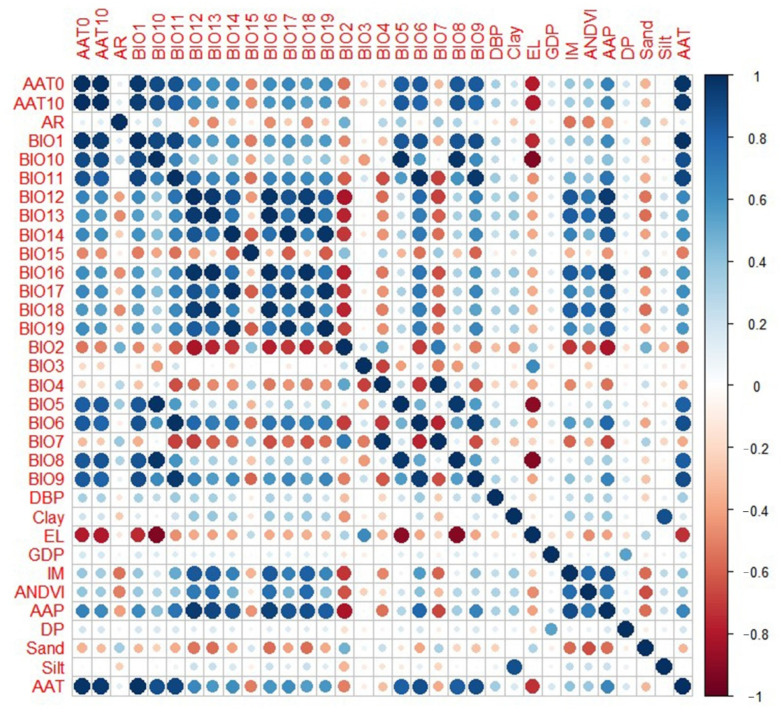
Diagnostics correlations with environmental factors.

As demonstrated in Figure 2, correlation diagnostics of environmental factors showed that most climate factors were highly correlated. To select the most predictive factors, we take the following selection criteria: if the correlated factors belong to the same dataset, such as the meteorological factors, bioclimatic factors, geographical factors, or socioeconomic factors, the factor that with the largest correlation coefficients will be kept for modeling, while the other correlated factors will be removed. Specifically, AAT had a strong correlation with AAT0 and AAT10 in the same data set, as did BIO1, BIO6, BIO9, and BIO11 for the bioclimatic factors. Because AAT correlates well with the other factors, AAT was selected for model training. The other factors were deleted. AAP had a strong correlation with annual precipitation BIO12, BIO13, and BIO16 for bioclimatic factors, and AAP was selected. BIO4 was strongly correlated with BIO7, hence the former was deleted. BIO19 had a strong correlation with BIO17 and BIO14, hence BIO19 was selected for model training. El, a geographic environmental variable, was related to BIO8. Since El was stronger than other climate factors, it was retained.

Finally, the factors included in the training model were four meteorological background factors, seven bioclimatic factors, seven geographic factors, and three socioeconomic factors, for a total of 21 environmental factors.

### 3.2. Performance of Single Ecological Niche Models and the Ensemble Model

#### 3.2.1. Performance of Model-Based Risk Prediction

**Figure 3:**
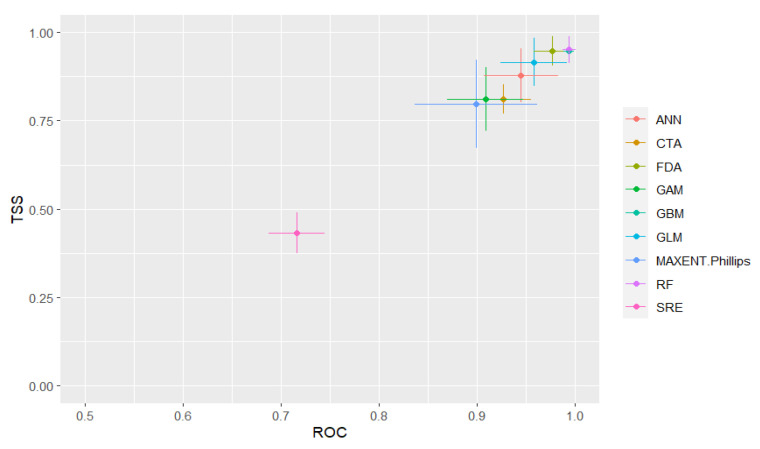
Two accuracy statistics (TSS and ROC) for 10 ecological niche models and the ensemble model.

ROC, kappa value, and TSS for 10 ecological niche models are presented in Figure 3. Based on mean ROC values (as measures of prediction accuracy), RF models were found to have the highest performance followed by GBM, FDA, MARS, GLM, GAM, ANN, CTA, MAXENT, and SRE models. Based on mean TSS values, RF models were found to have the best performance followed by GBM, FDA, MARS, GLM, GAM, CTA, ANN, MAXENT, and SRE models. Significant differences were observed between mean ROC (H = 60.363, *p* < 0.05), kappa (H = 54.447, *p* < 0.05), and TSS values (H = 53.894, *p* < 0.05) among all ecological niche models. Overall, the performance of the RF, GBM, FDA, MARS, and GLM models was satisfactory as a means by which to predict schistosomiasis transmission risk, with a mean ROC of >0.9 and a mean TSS of >0.85. Only the SRE model had poor performance, with a mean ROC of 0.726 ± 0.064, a mean kappa statistic of 0.457 ± 0.13, and a mean TSS of 0.452 ± 0.129. Two-way error scatter plots of mean ROC and TSS display the SRE model in the lower left and the GBM and RF models in the upper right of the coordinate system.

#### 3.2.2. Dominant Predictive Environmental Factors of the Ensemble Model

**Figure 4:**
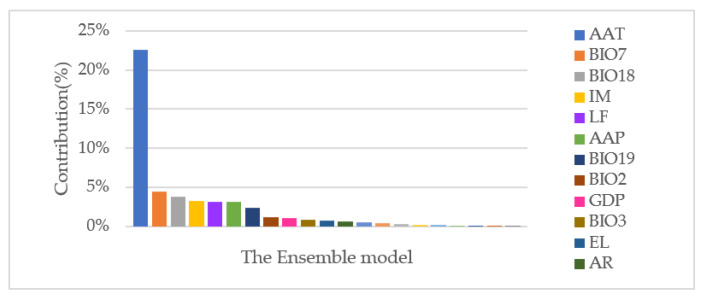
Environmental factors were dominant for risk prediction of the ensemble model.

The major environmental factors included in each ecological niche model were modeled (Figure 4). Environmental factors that made the greatest model contribution were average annual temperature (22.58%), temperature annual range (4.44%), precipitation in the warmest quarter (3.83%), index of moisture (3.30%), type of landform (3.15%), and average annual precipitation (3.12%).

### 3.3. Schistosomiasis Risk Prediction

**Figure 5:**
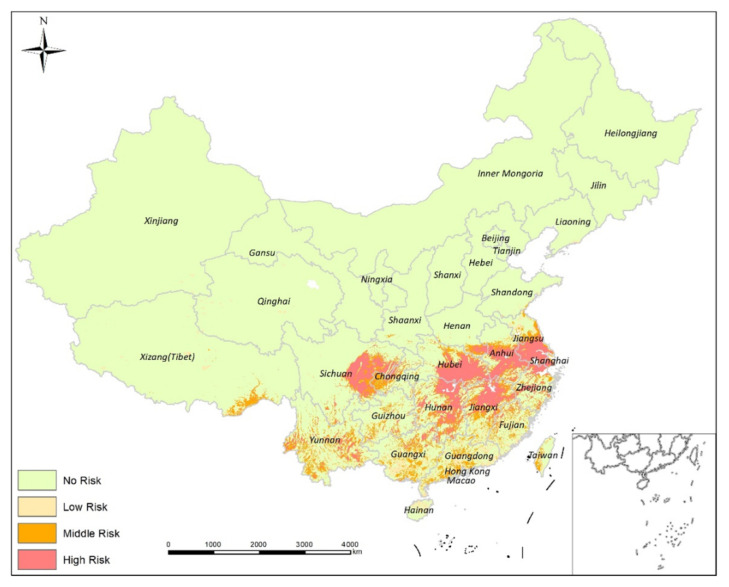
Mapping the risk of schistosomiasis transmission in China based on ensemble ecological niche modeling.

The ensemble model predicted the greatest risk for schistosomiasis transmission in China to be the middle and lower reaches of the Yangtze River, including southern Jiangsu province, western Shanghai prefecture, northern Zhejiang province, central Anhui province, the Poyang Lake areas of Jiangsu province, southern Hubei province, and the Dongting Lake areas of Hunan Province. Local risk area clustering was identified in central Sichuan province, with scattered risk areas predicted in central Yunnan province (Figure 5). Overall, the risk areas appeared to be clustered in the main schistosomiasis endemic area of China, with a scattered distribution pattern when evaluated at a larger scale. Schistosomiasis transmission risk prediction, using ecological niche models, identified an area infection risk of 10.96% for all of China, with a low risk of 4.55%, medium risk of 2.01%, and a high risk of 4.40%. There was no risk for 89.04% of China. Infection clusters were identified as high risk in southern Hubei Province, the Dongting Lake regions in northern Hunan province, northern parts, Poyang Lake regions of Jiangxi Province, the Yangtze River basin in central Anhui Province, and southern Jiangsu Province. Medium-risk areas were located in central Sichuan Province and local regions of Yunnan Province.

Mapping of the 12 endemic provinces of China identified to be at risk for schistosomiasis transmission. High-risk areas for schistosomiasis transmission were found in five provinces: Hubei (22.01%), Hunan (18.16%), Anhui (17.86%), Jiangsu (15.68%), and Jiangxi (7.04%). Provinces with medium risk for schistosomiasis transmission were: Hunan (21.35%), Sichuan (19.11%), Jiangxi (16.73%), Anhui (13.40%), and Hubei (11.66%). Multiple schistosomiasis transmission risk areas were found in Zhejiang Province. High-risk areas were also found in Shanghai Prefecture, medium and high-risk areas were found in Yunnan Province, and Fujian, Guangdong, and Guangxi had little or no risk for transmission.

## 4. Discussion

The epidemics of NTDs are sensitive in different ways to environmental and socioeconomic conditions [27,28,29], so the risk evaluation for NTDs requires data from multiple sources and multiple aspects, such as the geographical distributions of the pathogens, vectors, or host populations, as well as their related environmental determinants [30,31]. Ecological niche models integrate these datasets and utilize statistical approaches for predicting the potential distribution of vector species from survey-based observations [32]. In this study, climate, geographic, and socioeconomic factors were evaluated by developing an ensemble ecological niche model to predict the risk areas of schistosomiasis in mainland China.

Currently, the estimation of the value of area under the ROC curves is the primary method by which to evaluate the predictive performance of ecological niche models [33,34]. In this study, ROC in combination with Kappa and TSS were used to assess the predictive accuracy of models, with significant differences observed in the prediction accuracy of each of the 10 single ecological niche models. The result indicates most of the models worked well with higher performance except the SRE model. Although GLM, MARS, and GAM models were all generated based on a statistical regression algorithm, GLM and MARS models were found to produce more reasonable prediction results than GAM models. GBM, CTA, and FDA models, based on a classification algorithm, produced consistent predictions, with risk areas predicted for almost all sampling sites. With those models, there was no excessive risk area expansion, although a mild variation in the exact distribution of risk areas was observed. Among the three models based on machine learning algorithms, the RF model showed the highest prediction accuracy, with all high-risk areas accurately predicted within the main endemic foci of China. RF models also produced a reasonable shift from medium to low-risk areas, while the ANN model yielded loose predictive results. The MaxEnt model had poor prediction accuracy with many predicted risk areas observed to be outside of schistosomiasis endemic areas. These findings demonstrated that prediction accuracy by different ecological models may vary significantly and that uncertainty may exist in models created using the same algorithms.

The standard ecological niche models can generate uncertainties in the spatial predictions of disease transmission [35,36]. Although the accuracy of standard models can be achieved for risk prediction with the selected training and testing datasets, the predicted risk areas may not be in accord with the real-world situation. Multiple models using high index values have been shown to produce consistent prediction results, but the integration of these models will have better predictive accuracy and reduced spatial uncertainty [37].

In this study, the standard ecological niche models, which have high prediction accuracy, were integrated into an ensemble model. The results have shown that schistosomiasis high-risk regions were found to be predominantly located in southern Hubei Province, northern Hunan Province, central Anhui Province, northern Jiangxi Province, and southern Jiangsu Province, where the epidemic of schistosomiasis is under control. These predictions are in agreement with the distribution of transmission-controlled and -interrupted areas of China in 2018 [9].

The snail habitus sampled and included in this study was predominantly located in marshland, lake areas, and waterway networks. Models predicted potential high-risk areas in Shanghai and Zhejiang. This result indicated that the climate and ecological conditions in Shanghai and Zhejiang were environmentally suitable for schistosomiasis. Re-emergence of schistosomiasis is extremely likely upon the importation of *S. japonica*. Based on the results of the developed ensemble model, the risk of schistosomiasis transmission exists in the areas where the host snail is suitable for the local climate, environment, and socioeconomic conditions. The risk areas were classified, and high-risk areas were found to be mainly concentrated in the middle and lower reaches of the Yangtze River.

As a limitation of this study, the factors related to the disease intervention were not taken into account in the developed model. Therefore, the interpretation of prediction results was based on information from schistosomiasis control programs. For example, concerted control efforts over several decades have resulted in the elimination of schistosomiasis transmission in Shanghai and Zhejiang, where schistosomiasis was historically hyper-endemic. To improve predictive accuracy, future studies should consider the interventions that targeted schistosomiasis transmission, including examination and treatment of human schistosomiasis, bovine herd reduction, snail control with chemical treatment, and environmental modifications.

## 5. Conclusions

In this study, the transmission risk of schistosomiasis in mainland China was evaluated by developing an ensemble ecological niche model, which integrated 10 standard models. The risk areas predicted from this study provided a reference for schistosomiasis surveillance in China, which promoted the application of ecological niche modeling to the field of schistosomiasis transmission risk research. This study demonstrated that the proposed ensemble ecological niche model can be used for schistosomiasis transmission risk prediction and to inform schistosomiasis surveillance and control programs in risk areas. The inclusion of the latest artificial intelligence and machine learning algorithms in ecological niche models will broaden the field of schistosomiasis transmission risk research.

## Figures and Tables

**Table 1:** Environmental factors affecting the distribution of snails.

Variable Categories	Variable	Definition
Meteorological factors	AR	Aridity
	IM	Index of moisture
	AAP	Average annual precipitation
	AAT	Average annual temperature
	AAT0	≥0 °C annual accumulated temperature
	AAT10	≥10 °C annual accumulated temperature
Bioclimatic factors	BIO1	Annual mean air temperature
	BIO2	Monthly mean diurnal temperature range
	BIO3	Isothermality
	BIO4	Temperature seasonality
	BIO5	Maximum air temperature in the warmest month
	BIO6	Minimum air temperature in the coldest month
	BIO7	Temperature annual range
	BIO8	Mean air temperature in the wettest quarter
	BIO9	Mean air temperature in the driest quarter
	BIO10	Mean air temperature in the warmest quarter
	BIO11	Mean air temperature in the coldest quarter
	BIO12	Annual precipitation
	BIO13	Precipitation in the wettest month
	BIO14	Precipitation in the driest month
	BIO15	Precipitation seasonality
	BIO16	Precipitation in the wettest quarter
	BIO17	Precipitation in the driest quarter
	BIO18	Precipitation in the warmest quarter
	BIO19	Precipitation in the coldest quarter
Geographical factors	EL	Elevation
	LF	Type of landform
	LD	Type of land use
	Sand	Soil texture classified as sand
	Silt	Soil texture classified as silt
	Clay	Soil texture classified as clay
	ANDVI	Annual normalized difference vegetation index
Socioeconomic factors	DBP	The density of bovine populations
	GDP	Gross domestic product
	DP	Population density

**Table 2:** Ten standard ecological niche models.

Categories	Model	Definition
Environmental envelope algorithm	SRE	Surface range envelope
Statistical regression algorithm	GLM	Generalized linear models
	GAM	Generalized additive models
	MARS	Multivariate adaptive regression spline
Classification algorithm	GBM	Generalized boosted models model
	CTA	Classification tree analysis model
	FDA	Flexible discriminant analysis model
Machine learning algorithm	ANN	Artificial neural network model
	RF	Random forest model
	MaxEnt	Maximum entropy model

**Table 3:** Criteria for the measurement of prediction accuracy for ecological niche models.

Parameter	Failure	Poor	Fair	Good	Very Good
ROC	0.00–0.49	0.50–0.69	0.70–0.79	0.80–0.89	0.90–1.00
Kappa	−1.00–0.39	0.40–0.54	0.55–0.69	0.70–0.84	0.85–1.00
TSS	−1.00–0.39	0.40–0.54	0.55–0.69	0.70–0.84	0.85–1.00

## Data Availability

Not applicable.
